# Stevens-Johnson Syndrome Induced by Herbal Kadha

**DOI:** 10.7759/cureus.42407

**Published:** 2023-07-24

**Authors:** Varsha Y Lamture, Yashwant R Lamture, Punam Uke

**Affiliations:** 1 Department of Pharmacology, Jawaharlal Nehru Medical College, Datta Meghe Institute of Higher Education and Research, Wardha, IND; 2 Department of Surgery, Jawaharlal Nehru Medical College, Datta Meghe Institute of Higher Education and Research, Wardha, IND; 3 Department of Pediatrics, Jawaharlal Nehru Medical College, Datta Meghe Institute of Higher Education and Research, Wardha, IND

**Keywords:** emergency, anticonvulsant, kadha, ayurveda, hypersensitivity reaction

## Abstract

Stevens-Johnson syndrome (SJS) is a dreaded hypersensitivity reaction and a rare immune disorder. We present a Stevens-Johnson syndrome induced by herbal kadha, which may be the first case. A ten-year-old boy presented with massive sloughing, redness, oedematous skin, an oral ulcer, and an inability to feed or drink for two days. The present symptoms started after 12 hours of consuming herbal Kadha, given by a private practitioner in clinics where he was treated for fever. After not responding to earlier treatment, the patient was referred to the present Institute. The patient had a history of seizure disorder and had been on tablet phenytoin for seven months with no history of adverse reactions to it. He was treated in the intensive care unit. Fortunately, he responded to treatment and recovered fully. He received treatment in the form of immunoglobulin and steroids. Phenytoin and herbal kadha were withdrawn, and Clobazam was continued. Natural herbal medicines can develop severe adverse effects. Physicians should remain aware that drug interactions can likely be seen with drugs with a narrow therapeutic index combined with herbal preparations. Clinicians should do more research on the interaction between herbal and prescription medications.

## Introduction

Stevens-Johnson syndrome (SJS) is a life-threatening emergency due to a type four hypersensitivity reaction involving the skin, eyes, respiratory, renal, hepatic, and mucous membranes. The incidence is about 1 to 2 per 1,000,000 individuals annually [1]. SJS is caused by the ingestion of certain medicines, which include antibiotics, antiepileptics, and non-steroidal drugs. Viral infections and vaccines can provoke SJS [2]. Herbal treatments are used not only in India but also worldwide. Plant products have been used in India since the ancient period. So when allopathic drugs are prescribed, the patient continues the use of herbal products side by side, as it is considered safe. Adverse drug reactions are reported with the increasing use of herbal and allopathic medicines.

An example is Ginkgo biloba, which lowers the bioavailability of warfarin and alcohol. It precipitates bleeding with warfarin and psychotic effects like mania with alcohol [3]. So more studies are needed to understand adverse drug reactions caused by herb-drug interactions. As plant medicine is safe, patients and treating physicians do not register such cases in the pharmacovigilance center. It is important to notify any such issues to the pharmacovigilant center so that authorities will monitor public health safety about drugs.

Herbal kadha is a traditional herbal Indian preparation made of dry, spicy ingredients like peeled ginger, cloves, black pepper, basil leaves, and a cinnamon stick added to water, boiled to draw out the nutrients, and drunk twice to three times daily. It is recommended to boost immunity and came more into the limelight during the Corona pandemic [4].

We present a rare case of herbal kadha-induced SJS, a type 4 hypersensitivity reaction. We aim to make the physician aware of the potential side effects of such an herbal preparation, which claims to be safe.

## Case presentation

**Figure 1:**
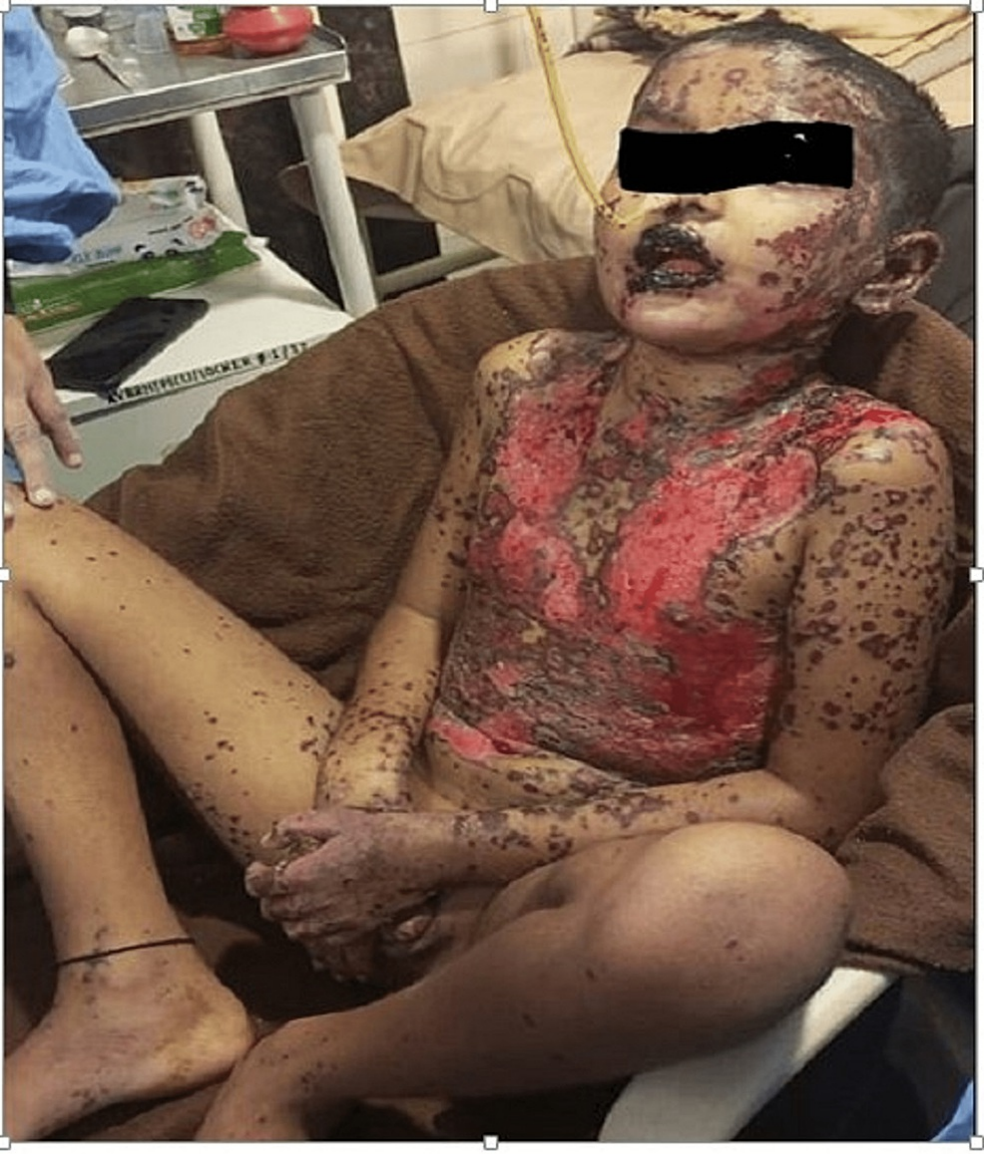
Presentation of child with massive sloughing, redness, oedematous skin, oral ulcer

A 10-year-old male child came in as a casualty with a history of massive sloughing of the skin (more than 10%), oedematous red and watering of both eyes, painful oral ulcers that were bleeding, difficulty swallowing, an increase in mouth secretion, an increase in respiratory rate, and fever (Figure 1).

His history reveals that he was admitted for a seizure disorder seven months ago. He responded to phenytoin, which has been continued to date. The patient was immediately shifted to the pediatric intensive care unit (PICU). On physical examination, the patient was dehydrated and had a temperature of 101 °C. The pulse rate was 122/min regular. All peripheral pulses were palpable. A bilateral wheeze was audible. The cardiovascular and abdominal examinations were within normal limits. The patient was immediately given intravenous fluids, a bronchodilator, antihistaminics, and hydrocortisone. After stabilizing the patient's vitals, we tried to gather detailed information about the cause of these allergic reactions. The parents gave us the history that the child had a fever for two days, so his parents took him to a nearby local practitioner, who gave him prepared herbal kadha without any label on it in the clinic itself. After 12 hours of ingestion of kadha, the parents observed reddish patches with itching all over the body, followed by skin sloughing and an oral ulcer. After 48 hours, the patient had difficulty swallowing and breathing, so the parents brought the child to the present center. Blood investigations showed that electrolytes were normal, hemoglobin was 12%, white blood cell count was 12,400, neutrophils were 87%, lymphocytes were 12%, eosinophils were 4%, and the erythrocyte sedimentation rate (ESR) was 62. Blood urea, serum glucose, and bicarbonate levels were within normal limits. He received appropriate treatment, including nutritional support. Systemic corticosteroids in the form of oral methylprednisolone were given for seven days with tapering doses. The patient was given intra-gastric feeding as she could not swallow food because of mouth ulcers. Intravenous immunoglobulins (IVIGs) of 0.75 g/kg once a day for four days were given. Third-generation cephalosporins and aminoglycosides were started to take care of the secondary infection. Maintenance of the mouth, skin, and eyes was done. The SCORTEN scale was applied on the first day of admission, which showed a score of 2 (Table 1).

**Table 1:** Severity-of-illness score (SCORTEN) The calculation of the score and its correlation with the fatality rate is as follows; the fatality rate is depicted in percentage. 0 to 1 = 3.2%, 2 = 12.1%, 3 = 35.3%, 4 = 58.3%, ≥5 = >90%.The score of the present case was 2 which comes to around 12%.

Risk factor	Score	
	0	1
Life span in years	Less than 40 years	More than 40 years
Associated malignancy	Not present	Present
Pulse rate/minutes	Less than 120	More than120
Blood urea (up to 28 mg/dL is normal)	normal	elevated
Damage body (surface in percentage)	Less than ten	More than ten
Bicarbonate levels (20 mEq is normal)	Decreased	Elevated
Glycemia (level 250 mg/dL is a critical level for measurement of score)	Decreased	Elevated

He was admitted for ten days of treatment and later discharged without complications. Phenytoin and herbal kadha were withdrawn, and Clobazam was added as an anticonvulsant. On follow-up after a month, the patient's vitals were stable, he was playful, faint scar marks on his chest were seen, and he had no seizures. The patient was advised to continue clobazam.

## Discussion

Any adverse drug reactions can present in mild to severe forms. Patients who usually need hospitalization are of severe form. SJS is an extreme form of hypersensitivity reaction and is likely drug-induced. The list of drug-induced reactions includes anticonvulsants, antibiotics, and anti-inflammatory agents [5].

Among anticonvulsants, phenytoin, sodium valproate, and carbamazepine are known to cause hypersensitivity reactions like SJS. Nakarmi et al., in their studies, showed that SJS frequently occurs within the first eight weeks of phenytoin therapy, and the risk increases if used in combination with other antiepileptics like sodium valproate or carbamazepine [6]. In the present case report, the patient received phenytoin alone for seven months, and no history of allergic reactions was recorded. The addition of Kadha resulted in SJS within 12 hours of ingestion.

A study by Pokladnikova et al. [7] on a long-term study of hypersensitivity reactions to herbal medicines in children showed that mixed herbal medicines containing zingiber rhizoma, cobots rhizoma, Asari herbal, epimedium deer antlers, ginseng, and camphor could trigger SJS. As the present case report received kadha without a label, we are unsure of the causative ingredient triggering SJS.

Lim et al. report an SJS case induced by traditional herbal medicine that is not associated with any other drugs. This Chinese medicine had ingredients like deer horn, ginseng, and camphor. The patient recovered after stopping herbal drugs, steroid administration, and supportive care [8]. The present case report has been on phenytoin for seven months with no adverse effect, but the addition of kadha stimulated SJS, which was dissimilar to the above case report.

Lim et al. presented a case series of four patients with SJS with a severe cutaneous adverse reaction to traditional herbal medicine alone. The authors suggested the significance of a physician's awareness of conventional medicine-induced adverse skin reactions [9]. This awareness should be seriously considered among fellow physicians when we add herbal medicine to prescriptions, considering it to be the safest.

The SCORTEN scale is a valuable tool for measuring the prognosis and severity of illness in various life-threatening conditions. Initially, it was used for severe burn injuries and toxic epidermal necrolysis. It was later used for Stevens-Johnson syndrome. SCORTEN is on a 0 to 7 scale. A score of over five has a bad prognosis and a 90% mortality rate. It includes risk factors like age, increased heart rate, detached or compromised body surface areas, raised blood urea nitrogen (BUN), serum glucose, and bicarbonate levels (Table 1). Roujeau et al. used the CORTEN score scale to determine patient mortality in SJS [10]. The SCORTEN score scale was used in the present case report. In the present case, the patient score was two as the heart rate was more than 120/min and the detachment of the body surface was more than 10%.

There are various suggestions for doing SCORTEN scores on multiple days, including days 1, 3, and 5. Jhanwar et al., in their study, used the CORTEN scale on the first, third, and fifth days of patients with SJS. Their results did not show any significant changes in scores. They concluded that the SCORTEN score on day 1 was enough to give us information about the prognosis [11]. The SCORTEN score was 2 in a present case report done on day 1 of admission.

There are no specific guidelines for managing SJS. Jacobsen et al. [12] pointed out that management protocols in every institution should include early diagnosis, removal of the triggering causative agents, supportive care, identification of complications, and prevention of subsequent events. Treatment options include immune-modifying drugs like local and systemic steroids, chemotherapeutic agents, and immunoglobulins. These treatment options were followed in the present case report. SJS induced by kadha alone has not been recorded despite extensive searches in English literature, even though it is consumed in every house in India. The reason could be ignorance that herbal medicine, including kadha, can trigger adverse drug reactions as it is considered safe. Patient visits both allopathic and ayurvedic physicians, so chances of drug interaction can take place. There needs to be more well-established research done on drug interactions between herbal and allopathic medicine. Therefore, monitoring and reporting such cases to the pharmacovigilance center is vital so that physicians can be aware of such events and timely intervention and notification can be done by regulatory authorities.

However, with the clinical history, herbal medicine is most likely the culprit drug in our case report, as the treating physician gave Kadha from her clinics, which caused SJS. On the other hand, the patient was on anticonvulsants for seven months without any hypersensitivity reactions. We take the responsibility to report this case because we think it is essential that clinicians be aware of the efficacy and adverse effects caused by herbal medicine, which is considered safe. Clinicians should also know how to classify cutaneous manifestations of drug hypersensitivity, considering the possibility of herbal medicine-induced drug reactions [13]. Also, since the present case has an early presentation of symptoms, the possibility of a viral infection, an anticonvulsant, or human leucocyte antigen (HLA) precipitating SJS cannot be denied. Ultimately, we will advise being vigilant and reporting such cases to the pharmacovigilance center to ensure public health safety and be aware of the consequences of herbal-drug interactions.

## Conclusions

From the above discussion, it could be concluded that natural herbal medicines can develop adverse effects. Patients who use herbal medicines do not inform their physician, thinking that because they are made of natural ingredients, they do not have a negative impact. More research should be done on the interaction between herbal and prescription medications. Physicians should remain aware that drug interactions can likely be seen with drugs with narrow therapeutic indices combined with herbal preparations.
